# Status of Sedentary Time and Physical Activity of Rural Residents: A Cross-Sectional Population-Based Study in Eastern China

**DOI:** 10.3389/fpubh.2022.838226

**Published:** 2022-04-14

**Authors:** Jiayuan Wang, Ya Wang, Mallikarjuna Korivi, Xi Chen, Rong Zhu

**Affiliations:** ^1^Second Clinical Medical College, Wenzhou Medical University, Wenzhou, China; ^2^School of Innovation and Entrepreneurship, Wenzhou Medical University, Wenzhou, China; ^3^Exercise Metabolism and Research Center, College of Physical Education and Health Sciences, Zhejiang Normal University, Jinhua, China; ^4^School of Sports Science, Wenzhou Medical University, Wenzhou, China

**Keywords:** sedentary behavior, physical activity levels, rural areas, adults, IPAQ-SF

## Abstract

**Background:**

The urbanization process may affect the lifestyle of rural residents in China. Limited information exists on the extent of sedentarism and physical activity (PA) level of rural residents in middle-income countries. This is the first survey on sedentary time (ST) and PA among rural residents in eastern China.

**Methods:**

This cross-sectional observational study randomly samples rural adults from Zhejiang Province in eastern China (*n* = 1,320). Participants' ST and PA levels were determined from the International Physical Activity Questionnaire Short Form through face-to-face interviews, and the influencing factors of PA levels were assessed through multi-class logistic regression analysis.

**Results:**

The findings showed that the daily ST of the participants ranged from 30 to 660 min, with a median of 240 min (P25, P75:120, 240 min), and 54.6% of participants were sedentary for 240 min or above. The daily ST in men, people aged 18 to 44 years, people with bachelors' degree and above, people working for government agencies or institutions, people with unmarried status, and people with an average income of < 2,000 Yuan was longer than that of other respective groups (*p* < 0.01). In contrast, the daily ST of people with hypertension or with patients with osteoporosis or osteopenia was less than that of normal people (*p* < 0.01). Additionally, 69.4% of participants generally had a low level of PA (LPA). Compared with those living in northern Zhejiang, people living in southern Zhejiang who were aged 18–44 years, had bachelor's degree or above, were farmers, and had household incomes below 10,000 Yuan per month were more likely to engage in LPA compared to people > 60 years, with high school or technical education levels or with junior college degrees, working in government agencies and institutions, and with household income above 10,000 Yuan per month (*p* < 0.05). Furthermore, there was no correlation between ST and PA levels.

**Conclusion:**

Most rural residents in the Zhejiang Province of eastern China had longer daily ST and a LPA. This was predominant in men, young people, highly educated people, unmarried people, and middle to high-income people. Health education programs should be targeted toward specific population groups to decrease the ST and increase PA.

## Introduction

Consistent with other countries, urbanization of rural areas in China has had a significant impact on the lifestyle of residents living in those areas (1). Developing rural areas from traditional agricultural societies to modern technology-dependent societies has not only led to changes in infrastructure and basic services (optimization and development of health and education), but also has led to changes in the living environment. Additionally, it has caused redistribution of time in life and has caused changes in the workstyles (e.g., from labor-intensive to sedentary work) (2). Due to the increased availability and use of modernized and automated agriculture machinery, rural residents and/or farmers are now free from having to perform manual labor (3). In addition, individual's sedentary behavior (SB) continues to increase due to an excessive use of multiple mobile electronic devices and playing computer games (4). People have lost their interest in participating in exercise programs, which results in a significant reduction in physical activity (PA) levels (5, 6). Several decades of economic reform and urbanization in China brought some unintended health consequences across Chinese society such as increased prevalence of overweight and obesity in adults, which was accompanied by decreased PA (7).

Currently, insufficient PA is a major risk factor for developing non-communicable diseases (NCDs), which has become the fourth leading cause of death in the world (8). PA helps in preventing overweight and obesity, alleviating stress, regulating mood, and improving mental health (9). Further, an adequate amount of PA is beneficial in preventing and treating NCDs, such as cardiovascular disease, stroke, diabetes, hypertension, and cancer (10). PA guidelines for Americans recommended that adults aged between 19 and 64 years should engage in 150 min of moderate-vigorous PA (MVPA) per week, which is commonly divided into five 30-min exercise sessions per week (11). Despite fulfilling this recommendation, some people still exhibit elevated cardiometabolic risk factors. In this case, such elevation might be associated with their SB rather than the amount of MVPA (12). Various strategies that can increase PA and reduce sitting behavior are considerable public health approaches to promote population health and wellbeing (13). The Chinese government publishes a national fitness plan every 5 years. The latest national fitness plan (2021–2025) promulgated in August 2021, which emphasizes the significance of regular PA in improving the health and quality of life of the population (14).

Zhejiang Province, located in the eastern coastal area of China, is one of the most developed provinces in China with rapid rural urbanization. Nevertheless, the SB and PA levels of rural residents in this province have not been elucidated. Thus, we explored the sociodemographic characteristics, ST, PA level, and chronic diseases of the rural residents in Zhejiang Province. The factors that could influence the PA level of individuals were further analyzed to aid in formulating health promotion policies.

## Methods

### Study Population and Sampling Design

This cross-sectional, observational study was conducted from November 2020 to January 2021 in Zhejiang Province. The representative samples were selected by a stratified, multi-stage, random cluster sampling strategy. In the first stage, all rural areas were identified from the statistical database of China's economic and social development (15). In the second stage, a total of 11 cities in Zhejiang Province were divided into the south (6 cities) and the north (5 cities) regions based on the geographic location and rural per capita disposable income. In the third stage, two cities each were randomly selected from the south and north regions. Then, 8 out of 40 counties were randomly selected from those 4 cities. In the next step, 14 out of 140 villages were randomly selected from those 8 counties. Approximately 100 residents were randomly selected from every village and conducted a face-to-face interview by the pre-trained investigators. Finally, a total of 1,351 respondents were considered, and the response rate was 97.7% (1,320/1,351).

### Measures

#### Participant's Characteristics

The survey questionnaire collected data on sociodemographic variables, which include age (18–44-, 45–59-, and ≥60-year groups) (16), gender (male or female), ethnicity (Han ethnicity or other), marital status (unmarried, married, divorced, widowed), education level (illiterate, primary school, junior high school, high school/technical, junior college, bachelor degree, and above), occupation (staff of agencies and institutions, corporate employees, commercial and service industry personnel, farmer, unemployed, retired personnel, and others), and average monthly household income (unclear, below 2,000, 2,000–4,999, 5,000–9,999, and more than 10,000 Yuan).

#### Assessment of ST and PA Level

Participants were asked to report their daily ST and PA levels in the past 7 days. The International Physical Activity Questionnaire Short Form (IPAQ-SF)—Chinese version with a test–retest reliability coefficient reaching 0.71–0.93 was used to obtain the data (17, 18). SB includes specific behaviors such as sitting or reclining or lying for reading, studying and watching television, and sitting at school or in public transportation (19). In IPAQ-SF, metabolic equivalents (METs) were used to measure the intensity. One MET is defined as the amount of oxygen consumed while sitting and resting. According to the criteria provided by the IPAQ scoring scheme, walking is set at 3.3 METs, moderate-intensity PA is set at 4 METs, and vigorous PA is set at 8 METs (20). The weekly level of an individual engaging in a certain intensity PA was the corresponding MET value of the PA × daily time (min/d) × weekly frequency (d/w).

The weekly MET value is calculated using the following formula:


$$\begin{array}{l} \text{WalkingPA}=3.3\text{METs}\times \text{min}\times \text{days},\\ \text{ModeratePA}=4\text{METs}\times \text{min}\times \text{days},\text{ and}\\ VigorousPA=8METs\times min\times days. \end{array}$$

Totally, three PA levels are classified according to the weekly PA frequency, intensity, and METs of the participants (20).

*High-level PA (HPA) needs to satisfy either of the following two criteria:*

Physical activity consisted of three intensities, 7 days per week, and the overall weekly PA is greater than or equal to 3,000 MET-min/week. HPA totals more than 3 days, and the weekly overall PA level is greater than or equal to 1,500 MET-min/week.

*Moderate-level PA (MPA) needs to meet any one of the following three criteria:*

At least 20 min of high-intensity PA per day for a total of more than 3 days; at least 30 min of moderate-intensity PA and/or walking per day for a total of more than 5 days; the three intensities of PA more than 5 days, and the weekly overall PA level is greater than or equal to 600 MET-min/week.

*Low-level PA (LPA):*

Physical activities of the participants that do not satisfy the above two levels (HPA or MPA) are considered as low-level PA and included in LPA.

#### Body Adiposity

The height (m) and weight (kg) of the participants were measured to calculate the body mass index (BMI, kg/m^2^). In China, the underweight was defined as BMI <18.5 kg/m^2^, the normal weight was defined as 18.5 kg/m^2^ ≤ BMI <24kg/m^2^, the overweight was defined as BMI ≥ 24 kg/m^2^, and obesity was defined as BMI ≥ 28 kg/m^2^, which were slightly different from the international standards (21, 22).

#### Hypertension

A well-trained medical doctor measured the participants' blood pressure three times while at rest. According to the hypertension standard guidelines, people who reported elevated blood pressures (SBP ≥ 140 mmHg and/or DBP ≥ 90 mmHg) or using antihypertensive drugs were considered as hypertensive patients (23). Then, all participants were divided into the hypertension group and the normal blood pressure group.

#### Osteoporosis

The calcaneal bone mineral density of the participants was measured by the portable bone densitometer (OsteoPro UBD 2002A). According to the World Health Organization's definition of osteoporosis, we categorized the participants into three groups, which include osteoporosis group (T value ≤ 2.5), osteopenia group (T value range from −2.5 to −1), and normal bone quality group (T value ≥ −1) (24).

### Statistical Analysis

All data collected were recorded two times in Epidata 3.1 to ensure its authenticity and accuracy. Descriptive statistics that includes counts, percentages, median (quartile), and 95% confidence intervals (CI) was used to describe the sample. Pearson's chi-square test and Fisher's exact test were used to assess the differences between the categorical variables. Kolmogorov–Smirnov test (K-S test) was used to verify whether a continuous variable satisfies a normal distribution, and data that did not satisfy a normal distribution were described by the median (quartile). The Mann–Whitney U or Kruskal–Wallis test was used to assess the differences among continuous variables. Multi-class logistic regression analysis was used to identify factors influencing the population's PA level and to verify whether the inclusion of independent variables was statistically significant. Using a two-sided test, statistical significance was set at the conventional level of *p* < 0.05, and all statistical analyses were performed using IBM SPSS Statistics for Windows, Version 25.0 (IBM, Armonk, NY, USA).

### Ethics Approval

All the procedures used in this study were reviewed and approved by the Wenzhou Medical University Human Research Ethics Committee (2021-010). All respondents were provided with a written informed consent form, which included the purposes of the study, medical items, and confidentiality agreements for personal information, before participating in the study.

## Results

### Sociodemographic Characteristics and Lifestyle of Participants

A total of 1,320 participants aged between 18 and 88 years (M = 52.3; SD = 14.2) participated in this study. Of those, 382 participants (28.9%) were aged between 18–44 years, 481 (36.4%) were aged 45–59 years, and 457 (34.6%) were aged 60 years and above. Most of the participants were of Han ethnicity (98.8%) and married (88.1%). More than half of the participants were women (62.8%) and farmers (55.0%), had junior high school and below education (65.3%), and had low PA levels (69.4%). The average income of the participants was below 5,000 Yuan (60.1%). The 50.5% and 49.5% of participants lived in northern Zhejiang and southern Zhejiang, respectively. Meanwhile, onsite measurements of height and weight, blood pressure, and bone mineral density were obtained from 1,275, 1,274, and 1,234 participants, respectively. Among them, 47.9% of participants were overweight and obese, 47.6% of participants had hypertension, and 29.9% had osteoporosis. Full details of participants' characteristics are presented in Table 1.

**Table 1:** Basic conditions of social demography and physique (*n* = 1,320), characteristics of daily ST.

	**Frequency (proportion)**	**Sedentary time (minutes/day)** **Median (Quartile)**	***p*** **-value**
Total	1,320	240 (120, 240)	
Gender			
Male	491 (37.2%)	240 (120, 240)	0.003^e^
Female	829 (62.8%)	240 (120, 240)	
Ethnicity			
Han nationality	1,304 (98.8%)	240 (120, 240)	0.460^e^
Other	16 (1.2%)	240 (180, 240)	
Age			
18–44	382 (28.9%)	240 (180, 360)^2**, 3**^	<0.001^d^
45–59	481 (36.4%)	180 (120, 240)^1**^	
Above 60	457 (34.6%)	180 (120, 240)^1**^	
Education			
Illiteracy	143 (10.8%)	180 (120, 240)^4**, 5**, 6**^	<0.001^d^
Primary education	379 (28.7%)	180 (120, 240)^4**, 5**, 6**^	
Junior high school education	340 (25.8%)	180 (120, 240)^4**, 5**, 6**^	
High school/technical	165 (12.5%)	240 (180, 300)^1**, 2**, 3**, 6**^	
Junior college	131 (9.9%)	240 (180, 360)^1**, 2**, 3**, 6*^	
Bachelor degree and above	162 (12.3%)	300 (240, 360)^1**, 2**, 3**, 4**, 5*^	
Profession			
Staff of agencies and institutions	199 (15.1%)	300 (240, 360)^2*, 3**, 4**, 5*, 6**^	<0.001^d^
Corporate employees	93 (7%)	240 (120, 330)^1*, 4**^	
Commercial and service industry personnel	66 (5%)	240 (120, 240)^1**^	
Farmer	726 (55%)	180 (120, 240)^1**, 2**, 7**^	
Unemployed	44 (3.3%)	240 (120, 345)^1*^	
Retired personnel	91 (6.9%)	120 (120, 240)^1**, 2**, 7**^	
Other	101 (7.7%)	240 (180, 360)^4**, 6**^	
Marital status			
Unmarried	74 (5.6%)	300 (240, 420)^2**, 4**^	<0.001^d^
Married	1,163 (88.1%)	240 (120, 240)^1**^	
Divorced	19 (1.4%)	240 (180, 360)	
Widowed	64 (4.8%)	240 (120, 240)^1**^	
Average monthly household income			
Below 2,000 Yuan	355 (26.9%)	180 (120, 240)^2*, 3**, 4**, 5**^	0.001^d^
2,000–4,999 Yuan	438 (33.2%)	240 (120, 240)^1*, 3**, 4*^	
5,000–9,999 Yuan	257 (19.5%)	240 (180, 300)^1**, 2**^	
Above 10,000 Yuan	125 (9.5%)	240 (120, 360)^1**, 2*^	
Not sure	145 (11%)	240 (120, 300)^1**^	
Living area			
Southern Zhejiang	654 (49.5%)	240 (120, 240)	0.327^e^
Northern Zhejiang	666 (50.5%)	240 (120, 240)	
PA level			
High-level	33 (2.5%)	180 (120, 270)	0.463^d^
Moderate-level	371 (28.1%)	240 (120, 240)	
Low-level	916 (69.4%)	240 (120, 240)	
Obesity^a^			
Normal and underweight people (BMI <24)	665 (52.2%)	240 (120, 240)	0.796^d^
Overweight people (24 ≦ BMI <28)	455 (35.7%)	240 (120, 240)	
Obese people (28 ≦ BMI)	155 (12.2%)	240 (120, 240)	
Hypertension ^b^			
Yes	607 (47.6%)	180 (120, 240)	<0.001^e^
No	667 (52.4%)	240 (120, 300)	
Bone condition^c^			
Normal	340 (27.6%)	240 (120, 360)^2**, **^	<0.001^d^
Osteopenia	525 (42.5%)	240 (120, 240)^1**^	
Osteoporosis	369 (29.9%)	240 (120, 240)^1**^	

1, 2, 3, 4, 5, 6The numbers before * refers to the comparison with the first/second/third/fourth/fifth/sixth group of the same variable.

a *Carried out in 1,275 subjects*.

b *Carried out in 1,274 subjects*.

c *Carried out in 1,234 subjects*.

d *Kruskal–Wallis test*.

e *Mann–Whitney U test*.

* *p < 0.05*;

** *p < 0.01*.

### Sedentary Time of Participants

**Figure 1:**
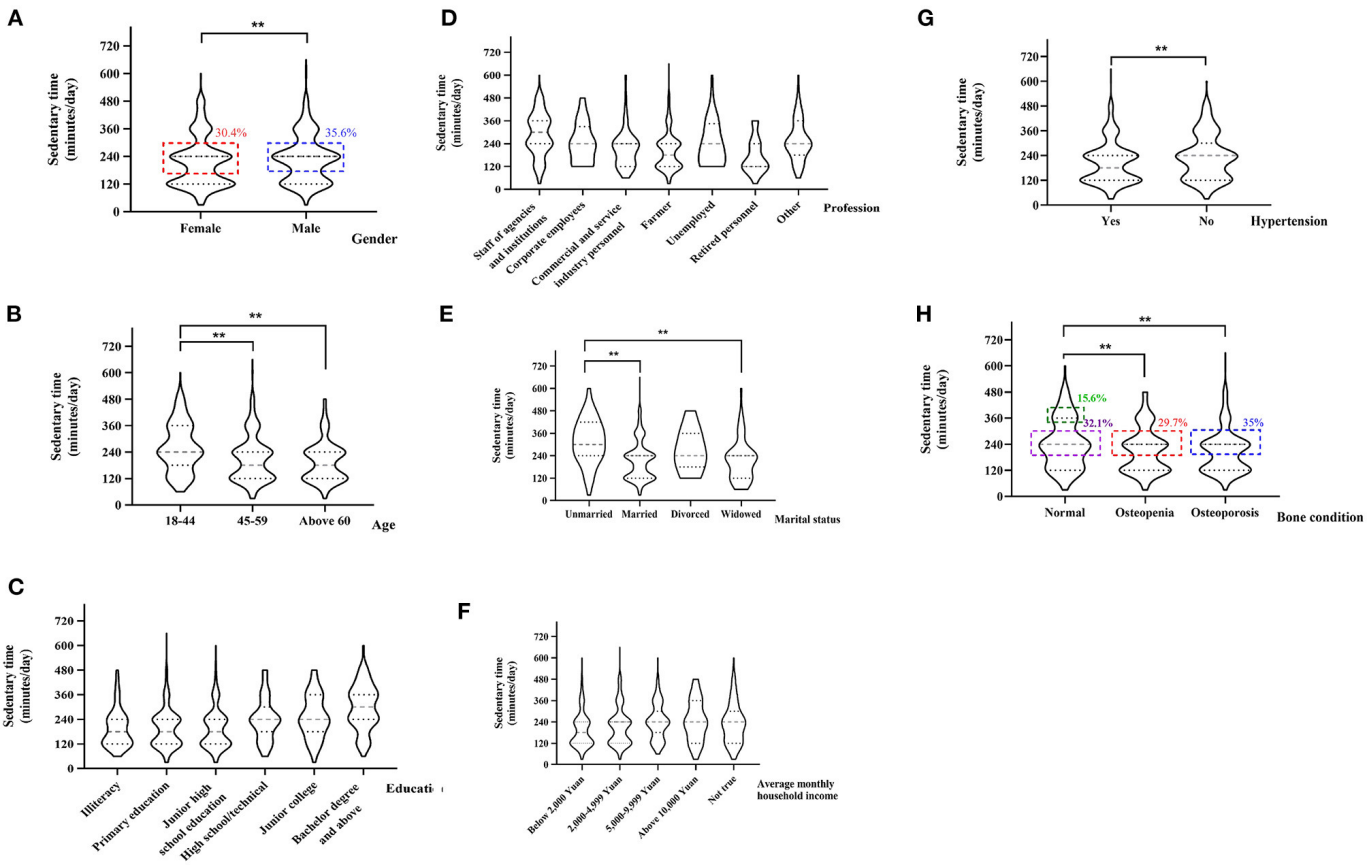
Violin diagram of the distribution of the ST of participants different in genders **(A)**, ages **(B)**, educations **(C)**, professions **(D)**, marital status **(E)**, average monthly household incomes **(F)**, blood pressure status **(G)**, and bone conditions **(H)**. **p < 0.01.

The daily ST of the participants ranged from 30 to 660 min with a median of 240 min (P25, P75:120 min, 240 min), and 54.6% of people were sedentary for ≥240 min. However, the daily ST of men was longer than the daily ST of women (*p* = 0.003), as men's ST distribution was more concentrated at 240 min (35.6%) when compared to women's ST distribution (30.4%) (Figure 1A). The association between ST and age revealed that the daily ST of participants in the 18–44-year age group was longer than the ST of participants in other age groups (*p* < 0.01). The median of the 18–44-year group was 240 min (p25, p75:180 min, 360 min), the median of 45–59-year group and above 60-year group was 180 min (p25, p75:180 min, 240 min) (Figure 1B).

In addition, the length of the daily ST significantly differed among people with different education levels, occupations, marital status, and household monthly incomes (all *p* < 0.01). Longer daily ST was found among people with higher educational qualifications. To be specific, people with bachelor's degree and above had the longest daily ST (median: 300 min) compared to the other education levels (*p* < 0.01) (Figure 1C). The participants working for government agencies and institutions had the longest daily ST (median: 300 min) when compared to participants with other occupations (*p* < 0.01). Besides, farmers and unemployed people had lower daily ST (median: 180 and 120 min) when compared to participants of other occupation groups (*p* < 0.01) (Figure 1D). As shown in Figure 1E, unmarried people had longer daily ST (median: 300 min) when compared to married people (*p* < 0.01). Further, residents with lower household incomes had lower levels of daily ST. Specifically, residents with an average income of <2,000 Yuan (median: 180 min) had significantly lower ST than residents in other income groups (median: 240 min) (Figure 1F).

It is interesting to note that the daily ST of people with hypertension (median: 180 min) was shorter than that of people without hypertension (median: 240 min) (*p* < 0.01, Figure 1G). Furthermore, the daily ST of people with osteoporosis or osteopenia (35%/29.7% focused on 240 min) was shorter than that of people with normal bone density (32.1% focused on 240 min and 15.6% focused on 360 min) (*p* < 0.01, Figure 1H). Additionally, we found no difference in ST among different PA levels (*p* = 0.463 > 0.05) (Table 1).

Among various SBs, screen time (watching TV, using computers, mobile phones, and other electronic devices) was exhibited by the greatest percentage of participants (83.3%) followed by communication or chatting with neighbors and friends (36.2%). Only 17.1% of participants were involved in recreational activities, such as playing mah-jong and playing cards (Table 2).

**Table 2:** Daily SBs of rural residents.

**Sedentary project**	**Number of people (***n*** = 1,320)**	**Percentage**
Watching TV, computers, mobile phones and other electronic products	1,100	83.3%
Playing mahjong, poker	226	17.1%
Chatting with neighbors and friends	478	36.2%
Reading, reading newspapers, listening to the radio	176	13.3%
Other	64	4.8%

### PA Level of Participants

Physical activity levels of participants are shown in Table 3. Our findings revealed that majority of participants (69.4%) had low levels of PA, and only 30.6% of participants achieved moderate-to-high level PA. The three PA levels were significantly different among age groups (χ^2^ = 28.89, *p* < 0.001), education levels (χ^2^ = 33.78, *p* < 0.001), profession (χ^2^ = 35.40, *p* < 0.001), household income (χ^2^ = 31.73, *p* < 0.001), and living area (χ^2^ = 51.54, *p* < 0.001). These differences were analyzed by multi-class logistic regression (Table 4).

**Table 3:** Analysis of the PA level of residents in rural areas of Zhejiang Province.

	**PA level**			**Chi-square value**	***p*** **value**
	**High (*n* = 33)**	**Moderate (*n* = 371)**	**Low (*n* = 916)**		
Total	33 (2.5%)	371 (28.1%)	916 (69.4%)		
Gender					
Male	16 (3.3%)	136 (27.7%)	339 (69%)	χ^2^ = 1.86	0.395
Female	17 (2.1%)	235 (28.3%)	577 (69.6%)		
Nationality					
Han ethnicity	32 (2.5%)	368 (28.2%)	904 (69.3%)	χ^2^ = 1.49	0.475
Other	1 (6.3%)	3 (18.8%)	12 (75%)		
Age					
18–44	18 (4.7%)	81 (21.2%)	283 (74.1%)	χ^2^ = 28.89	**<0.001**
45–59	11 (2.3%)	164 (34.1%)	306 (63.6%)		
Above 60	4 (0.9%)	126 (27.6%)	327 (71.6%)		
Education					
Illiteracy	1 (0.7%)	30 (21%)	112 (78.3%)	χ^2^ = 33.78	**<0.001**
Primary education	4 (1.1%)	97 (25.6%)	278 (73.4%)		
Junior high school education	7 (2.1%)	111 (32.6%)	222 (65.3%)		
High school/technical	7 (4.2%)	57 (34.5%)	101 (61.2%)		
Junior college	4 (3.1%)	42 (32.1%)	85 (64.9%)		
Bachelor degree and above	10 (6.2%)	34 (21%)	118 (72.8%)		
Profession					
Staff of agencies and institutions	9 (4.5%)	62 (31.2%)	128 (64.3%)	χ^2^ = 35.4	**< 0.001**
Corporate employees	8 (8.6%)	25 (26.9%)	60 (64.5%)		
Commercial and service industry personnel	1 (1.5%)	19 (28.8%)	46 (69.7%)		
Farmer	11 (1.5%)	190 (26.2%)	525 (72.3%)		
Unemployed	1 (2.3%)	17 (38.6%)	26 (59.1%)		
Retired personnel	2 (2.2%)	36 (39.6%)	53 (58.2%)		
Other	1 (1.0%)	22 (21.8%)	78 (77.2%)		
Marital status					
Unmarried	5 (6.8%)	19 (25.7%)	50 (67.6%)	χ^2^ = 7.81	0.252
Married	28 (2.4%)	329 (28.3%)	806 (69.3%)		
Divorced	0 (0%)	5 (26.3%)	14 (73.7%)		
Widowed	0 (0%)	18 (28.1%)	46 (71.9%)		
Average monthly household income					
Below 2,000 Yuan	4 (1.1%)	83 (23.4%)	268 (75.5%)	χ^2^ = 31.73	**<0.001**
2,000–4,999 Yuan	15 (3.4%)	112 (25.6%)	311 (71%)		
5,000–9,999 Yuan	6 (2.3%)	72 (28%)	179 (69.6%)		
Above 10,000 Yuan	6 (4.8%)	54 (43.2%)	65 (52%)		
Not sure	2 (1.4%)	50 (34.5%)	93 (64.1%)		
Living area					
Southern Zhejiang	4 (0.6%)	141 (21.6%)	509 (77.8%)	χ^2^ = 51.54	**<0.001**
Northern Zhejiang	29 (4.4%)	230 (34.5%)	407 (61.1%)		
Smoking status					
Smoking every day	7 (3.6%)	49 (25.3%)	138 (71.1%)	χ^2^ = 3.31	0.77
Occasionally smoking	1 (1.6%)	18 (29%)	43 (69.4%)		
Used to smoke, now quit	1 (1.5%)	23 (34.3%)	43 (64.2%)		
Never smoked	24 (2.4%)	281 (28.2%)	692 (69.4%)		
Drinking status					
Drinking every day	0 (0%)	24 (24.2%)	75 (75.8%)	χ^2^ = 6.45	0.375
Occasionally drinking	10 (2.5%)	109 (27.6%)	276 (69.9%)		
Used to drink, now abstain	2 (3.4%)	12 (20.3%)	45 (76.3%)		
Never drank	21 (2.7%)	226 (29.5%)	520 (67.8%)		
Obesity^a^					
Normal and underweight people	14 (2.1%)	181 (27.2%)	470 (70.7%)	χ^2^ = 5.15	0.273
Overweight people	14 (3.1%)	140 (30.8%)	301 (66.2%)		
Obese people	5 (3.2%)	36 (23.2%)	114 (73.5%)		
Hypertension^b^					
Yes	11 (1.8%)	172 (28.3%)	424 (69.9%)	χ^2^ = 2.34	0.311
No	21 (3.1%)	184 (27.6%)	462 (69.3%)		
Bone condition^c^					
Normal	8 (2.4%)	102 (30%)	230 (67.6%)	χ^2^ = 6.50	0.165
Osteopenia	17 (3.2%)	152 (29%)	356 (67.8%)		
Osteoporosis	6 (1.6%)	89 (24.1%)	274 (74.3%)		

a *Carried out in 1,275 subjects*.

b *Carried out in 1,274 subjects*.

c *Carried out in 1,234 subjects. The bold values indicates significant p values (< 0.05)*.

**Table 4:** Multivariate logistic regression analysis of factors affecting the level of PA of rural residents.

	**Low-level PA vs. High-level PA**		**Moderate-level PA vs. High-level PA**		**Low-level PA vs. Moderate-level PA**	
	**B**	**OR (95%CI)**	**B**	**OR (95%CI)**	**B**	**OR (95%CI)**
Intercept	2.289		2.091		0.198	
Southern Zhejiang	1.913	6.770 (2.238–20.483) **	1.218	3.379 (1.100–10.378) *	0.695	2.003 (1.521–2.639) **
Northern Zhejiang	Reference	.	Reference	.	Reference	.
18–44	−0.573	0.564 (0.119–2.676)	−1.537	0.215 (0.044–1.045)	0.964	2.623 (1.616–4.257) **
45–59	−0.550	0.577 (0.153–2.176)	−0.622	0.537 (0.141–2.048)	0.072	1.075 (0.771–1.498)
Above 60	Reference	.	Reference	.	Reference	.
Illiteracy	0.968	2.632 (0.200–34.625)	1.113	3.042 (0.219–42.327)	−0.145	0.865 (0.380–1.972)
Primary education	0.745	2.107 (0.370–12.011)	1.138	3.122 (0.515–18.928)	−0.393	0.675 (0.328–1.389)
Junior high school education	0.624	1.866 (0.462–7.548)	1.29	3.633 (0.838–15.750)	−0.666	0.514 (0.262–1.008)
High school/technical	0.034	1.035 (0.303–3.529)	0.857	2.355 (0.647–8.573)	−0.822	0.439 (0.232–0.832) *
Junior college	0.805	2.237 (0.617–8.112)	1.484	4.412 (1.149–16.938) *	−0.679	0.507 (0.280–0.917) *
Bachelor degree and above	Reference	.	Reference	.	Reference	.
Other	1.370	3.934 (0.437–35.392)	1.168	3.215 (0.344–29.998)	0.202	1.224 (0.690–2.169)
Staff of agencies and institutions	−0.256	0.775 (0.211–2.843)	0.402	1.495 (0.391–5.715)	−0.657	0.518 (0.296–0.907) *
Corporate employees	−0.663	0.515 (0.164–1.622)	−0.710	0.492 (0.148–1.636)	0.047	1.048 (0.590–1.862)
Commercial and service industry personnel	0.781	2.185 (0.255–18.702)	0.833	2.301 (0.261–20.296)	−0.052	0.950 (0.520–1.733)
Unemployed	0.087	1.091 (0.127–9.375)	0.382	1.465 (0.167–12.862)	−0.295	0.745 (0.377–1.472)
Retired personnel	0.165	1.180 (0.237–5.867)	0.172	1.188 (0.236–5.987)	−0.007	0.993 (0.606–1.627)
Farmer	Reference	.	Reference	.	Reference	.
Not sure	0.637	1.890 (0.340–10.513)	0.282	1.326 (0.234–7.511)	0.355	1.426 (0.826–2.463)
Below 2,000 Yuan	0.665	1.945 (0.439–8.624)	−0.299	0.741 (0.164–3.353)	0.965	2.624 (1.583–4.349) **
2,000–4,999 Yuan	0.131	1.140 (0.402–3.234)	−0.653	0.521 (0.179–1.513)	0.784	2.190 (1.384–3.466) **
5,000–9,999 Yuan	1.026	2.789 (0.826–9.415)	0.355	1.426 (0.413–4.923)	0.671	1.956 (1.212–3.157) **
Above 10,000 Yuan	Reference	.	Reference	.	Reference	.

*There is no collinearity between the independent variables*.

** *p < 0.01*.

* *p < 0.05. OR (95%CI) for statistically significant variables are shown in bold*.

By considering HPA as the control group, the southern Zhejiang residents were 6 times more likely to engage in LPA (OR: 6.77, 95%CI: 2.238–20.483, *p* < 0.01) and 3 times more likely to engage in MPA (3.379, 1.100–10.378, *p* < 0.05) when compared to the northern Zhejiang residents. In addition, people with a junior college degree were 4 times more likely to engage in MPA than those with a bachelor's degree or above (4.412, 1.149–16.938, *p* < 0.05).

Considering MPA as the control group, residents of southern Zhejiang were only 2 times likely to engage in LPA compared to the northern Zhejiang residents (2.003, 1.521–2.639, *p* < 0.01). People aged between 18 and 44 years were 2 times more likely to engage in LPA compared with people over 60 years old (2.623, 1.616–4.257, *p* < 0.01).

Compared to the participants with bachelor college degree or above, participants with high school or technical education levels are only 43.9% likely to engage in LPA (0.439, CI = 0.232–0.832, *p* < 0.05), while 50.7% junior college degree holder are likely to engage in LPA (0.507, CI = 0.280–0.917, *p* < 0.05). On the other hand, 51.8% of staff working for government agencies and institutions are more likely to engage in LPA than farmers (0.518, CI = 0.296–0.907, *p* < 0.01). Noteworthy, participants with household income below 2,000 Yuan, 2,000–4,999 Yuan, and 5,000–9,999 Yuan per month are 2 times more likely to engage in LPA when compared to the participants with household income above 10,000 Yuan per month (Table 4).

These findings indicate that the major obstacles for PA are the lack of time (61.1%), the lack of interest (18.3%), the lack of fitness for PA (11.7%), less economic income (4.8%), fear of ridicule (2%), and other reasons (15.1%) (Table 5).

**Table 5:** Barriers to participation in physical activities by rural residents.

**Obstacle factor**	**Number of people (***n*** = 1,320)**	**Percentage**
Not interested	242	18.3%
Fear of ridicule	26	2%
No fit for PA	154	11.7%
Lack of time	806	61.1%
Low income	64	4.8%
Other	199	15.1%

## Discussion

Our findings identified the levels of ST and PA of rural residents of Zhejiang province and demonstrated the key variables responsible for the longer SB and shorter PA. It has been suggested that SB should be recorded using SB frequency, interruptions in SB, duration of each SB, and type of SB; this is similar to the measurement of PA using frequency, intensity, time, and type (25). The duration of each SB used in our study indicates the time spent in SB lasted for half an hour or above without changing the posture. In our study, the ST of participants in Zhejiang province is almost 4 h, which is similar to the ST (3.91 ± 2.06 h) of people in four counties of Shanxi and Chongqing provinces in China (26), while the ST of rural residents in other countries, such as Southern Brazil, Poland, and America (Texas), is generally 4–6 h (6, 27, 28).

To improve health and fitness, the Canadian's 24-h Movement Guidelines (29) and Physical Activity Guidelines for Americans (PAG) (30) recommended that the adult population should reduce their ST (sit less) throughout the day (8 h or less), include no more than 2–3 h of screen time, and break up long periods of sitting. Consistent with the previous studies from Henan rural residents in China, rural adults covering 31 provinces across China, and a review study with 10 countries over the world (31–33), men sit longer than women implying that rural women bear more housekeeping responsibilities than men, while men spent more time playing mah-jong and poker. Older people, especially those aged above 60 years, tend to be more sedentary than younger people in the previous studies (34–36). However, adults aged between 18 and 44 years had significantly longer ST than the other age groups in our study, which may be related to the Chinese traditional habit of people above 45 years of age willing to do more housework duties to take care of their posterity.

Longer ST of participants in our study was associated with the level of education, which is similar to earlier reports (37–40). People working for government agencies or institutions also had longer daily ST than those with other occupations including farming, unemployment, and corporate or commercial service industry occupations, who had low education levels, low socioeconomic status, the lack of use of electronic equipment (41), and tendency to engage in more active or less sedentary jobs (40). In addition, there was a negative correlation between rural residents' educational level and age. Precisely, 75.9% of people below primary school education were aged above 60 years, and 72% of people below the junior high school level were aged above 45 years, which may be the reason why the daily ST of adults aged ≥45 years was less than that of adults aged ≥18–44 years. We found that people with low education levels spent ST watching TV, talking with friends, playing mah-jong and poker, reading, listening, and other activities, which was consistent with the previous studies conducted on the general population (36, 42). Our study showed that unmarried people spend more ST than other groups, which was similar to the findings of the previous research on SB (43, 44). One possible reason was that marriage can act as a protective factor against unhealthy behaviors, as unmarried people tend to be sedentary for long periods, mainly the impact of not having a partner to hold them accountable for their unhealthy behavioral habits (45). This suggested that a marriage or long-term relationship contributes significantly to an individual's quality of life (44). We found that the average household income level was also a factor affecting the SB of rural residents, and the residents with higher income levels tended to have higher levels of SB (6, 46, 47). The possible reason for this is that rural residents of wealthy households are less likely to engage in strenuous work and daily household chores, which increases the risk of being sedentary. The lower-income families, especially peasant families, are often engaged in some labor involved job which seldom allow for these groups sit for long periods.

Although the previous research showed that SB was associated with an increased risk of hypertension (48), we found that the participants with hypertension or osteopenia or osteoporosis spent less daily ST than the participants with normal blood pressure or normal bones mass. The actual causes of hypertension are multifactorial and include genetical, behavioral, and environmental factors (49). On the other hand, there is no literature to show that patients with hypertension have less ST than people with normal blood pressure. Participants with hypertension may be aware of the risk of hypertension and are actively taking actions to reduce ST. In addition, the increase of blood pressure and the decrease of bone mass are related to the increase of age (50), while in our study, older people had less ST than younger people. Consistent with the previous studies (51), we found that there was no difference in ST among rural residents with different BMI. The available evidence does not fully support an association between SB and obesity in the adult population, as there is not strong enough evidence that elevated levels of ST cause longitudinal increases in BMI.

There is a relationship between the socioeconomic status of a country and levels of PA. Wang et al. (52) clearly stated that people tend to engage in more PA in economically advanced regions in China. Therefore, even in the same country with the same governance and healthcare system, there are geographical differences in the level of PA among different groups of people (53). We observed that the residents in the southern part of Zhejiang are more inclined to engage in LPA rather than HPA and MPA when compared to residents in the northern part of Zhejiang. Such occurrence might be due to the higher economic level, public sports service facility, and national health publicity in Northern Zhejiang. This finding may help researchers to further explore rural residents' awareness of PA in different living areas, as well as develop culturally adapted public health recommendations and intervention measures.

With an increase in age, it is inevitable that the decline of human physical function leads to decreased PA levels, and it is difficult for the elderly to achieve higher PA levels (26, 54). However, we observed that when compared to those aged above 60 years, adults aged 18–44 years were more inclined to engage in LPA rather than MPA in the rural areas. These findings agree with a previous study that the PA level of older adults was not necessarily lower than that of young adults (55), and the PA of older adults increased with increasing age (56–58). These results imply that young people may spend more time on their work and less time on PA. We observed that a group fitness activity in rural, such as square dancing, usually attracts older adults, more specifically older women who like to walk and dance together (59) and may be a considerable reason why women in rural areas spent less sitting time than men.

In rural areas of Zhejiang Province, compared with farmers, people working in public institutions were more inclined to engage in MPA rather than LPA. This is because government organizations and institutions responded to the national fitness plan issued by the state. Although the staff of government agencies and public institutions spend 1/3 of their day on sedentary work, the work unit could actively carry out the sports activities and encourage people to participate in sports competitions. To pass the national physical fitness test, some institutions not only implemented national fitness education but also provided a suitable environment to promote employees exercising (60). Farmers tended to do LPA, such as repeatedly standing upright and bending the waist to harvest crops. Farmers did not engage in enough PA to maintain their health and ability in agricultural activities (6), which is not a healthy practice of physical exercise (61). Therefore, it is necessary to encourage rural residents to engage in PA regularly.

In agreement with our findings, Zhao et al. also reported that education level and average monthly income are the key factors that affect physical exercise (26). However, we observed that people with high school or technical education or junior college are more inclined to engage in MPA than those with bachelor's degree and above. The possible reason is that people with a high school or technical or junior college education are more likely to be older than people with a bachelor's degree or above. Furthermore, people with an average monthly income of <4,999 Yuan are more likely to engage in LPA, which means that people with different economic incomes will participate in different levels of PA (9). Therefore, the government should pay attention to low-income rural residents and intervene by publicizing health awareness and sports literacy to improve people's positive understanding of the importance of participating in sports activities.

In this study, we found no correlation between ST and the intensity of PA participation in rural residents. Although sufficient moderate-to-vigorous intensity PA may only compensate the health risks of high ST to a certain degree (62), the association between PA and ST and health outcomes are independent of each other. Even if an individual's PA level reaches a moderate level or the recommended index, sitting for a long time can increase the risk of chronic diseases (63, 64). SB and lack of PA have been identified as independent health risk factors for the development of NCDs, including metabolic syndrome, overweight or obesity, hypertension, and cognitive impairment (65, 66).

The health status of residents in rural areas in Zhejiang cannot be ignored and will be improved by interventions that promote increasing the level of PA and reducing ST. Despite the rapid progress of rural urbanization, rural economic development was still backward compared with cities (31), which made rural residents more vulnerable to risks due to lack of PA and long-term ST (3, 6, 31). At present, Chinese residents have realized that PA can improve health, but they still do not understand the health risks caused by SB (67). Therefore, it is an urgent need to promote a healthy lifestyle to rural residents (68). Most rural residents in Zhejiang Province admitted that they have no spare time to participate in PA, which reminds us that time management should also be considered when providing exercise advice to them.

### Limitations and Strengths

First, as this is a cross-sectional study design, causality between influencing factors and PA level or SB could not be assessed. Second, it was somewhat difficult to assess the PA and ST precisely due to self-measurement of ST and PA. Third, potential confounding factors in multivariate analysis and unmeasured factors may affect our findings. In addition, despite the same wording, questions on PA and SB may be understood or interpreted differently according to a person's culture, gender, or social background. Nonetheless, our findings have been useful for designing effective PA programs in rural communities. This study has various strengths such as the use of objective instruments to measure hypertension and bone mineral density, which offers more accurate data than other self-reported tools. An important strength is the relatively large sample size collected from different rural areas of Zhejiang Province. Moreover, in response to the scarcity of studies of this type in the Chinese rural population, this study offers new insight to better understand ST and PA levels.

## Conclusion

This is the first population-based cross-sectional study that investigated the ST and PA levels of rural residents in eastern China. The findings revealed that most Chinese, especially men, young people, highly educated people, unmarried people, and middle and high-income people, had longer daily ST and a low level of PA (LPA). Nevertheless, the ST of patients with hypertension or osteopenia or osteoporosis was less than normal people. Therefore, we suggest that health education programs should accurately target different populations to improve the effectiveness of PA and reduce the SB in rural residents.

## Data Availability Statement

The original contributions presented in the study are included in the article/Supplementary Material, further inquiries can be directed to the corresponding author/s.

## Ethics Statement

The studies involving human participants were reviewed and approved by Wenzhou Medical University Human Research Ethics Committee (2021-010). The patients/participants provided their written informed consent to participate in this study.

## Author Contributions

JW, RZ, and YW: data collection. JW and RZ: data analysis and original draft preparation. JW, RZ, MK, YW, and XC: review and editing. RZ: funding acquisition. All authors have read and agreed to the published version of the manuscript.

## Funding

This study was supported by the National Social Science Fund of China (18BTY105).

## Conflict of Interest

The authors declare that the research was conducted in the absence of any commercial or financial relationships that could be construed as a potential conflict of interest.

## Publisher's Note

All claims expressed in this article are solely those of the authors and do not necessarily represent those of their affiliated organizations, or those of the publisher, the editors and the reviewers. Any product that may be evaluated in this article, or claim that may be made by its manufacturer, is not guaranteed or endorsed by the publisher.
